# Bench-to-bedside: Feasibility of nano-engineered and drug-delivery biomaterials for bone-anchored implants and periodontal applications

**DOI:** 10.1016/j.mtbio.2022.100540

**Published:** 2022-12-30

**Authors:** Marcel F. Kunrath, Furqan A. Shah, Christer Dahlin

**Affiliations:** a Department of Biomaterials, Institute of Clinical Sciences, Sahlgrenska Academy at University of Gothenburg, P.O. Box 412, SE 405 30, Göteborg, Sweden; b Department of Dentistry, School of Health and Life Sciences, Pontifical Catholic University of Rio Grande Do Sul (PUCRS), Porto Alegre, Brazil

**Keywords:** Biomaterials, Clinical translation, Nanotechnology, Pharmacological devices, Multifunctional materials, Drug release

## Abstract

Nanotechnology and drug-release biomaterials have been thoroughly explored in the last few years aiming to develop specialized clinical treatments. However, it is rare to find biomaterials associated with drug delivery properties in the current dental market for application in oral bone- and periodontal-related procedures. The gap between basic scientific evidence and translation to a commercial product remains wide. Several challenges have been reported regarding the clinical translation of biomaterials with drug-delivery systems (BDDS) and nanofeatures. Therefore, processes for BDDS development, application in preclinical models, drug delivery doses, sterilization processes, storage protocols and approval requirements were explored in this review, associated with tentative solutions for these issues. The diversity of techniques and compounds/molecules applied to develop BDDS demands a case-by-case approach to manufacturing and validating a commercial biomaterial. Promising outcomes such as accelerated tissue healing and higher antibacterial response have been shown through basic and preclinical studies using BDDS and nano-engineered biomaterials; however, the adequate process for sterilization, storage, cost-effectiveness and possible cytotoxic effects remains unclear for multifunctional biomaterials incorporated with different chemical compounds; then BDDSs are rarely translated into products. The future benefits of BDDS and nano-engineered biomaterials have been reported suggesting personalized clinical treatment and a promising reduction in the use of systemic antibiotics. Finally, the launch of these specialized biomaterials with solid data and controlled traceability onto the market will generate strong specificity for healthcare treatments.

## Highlights

• Drug-delivery biomaterials have shown promising advances for clinical treatments in preclinical studies.
• The presence of commercial drug-delivery dental biomaterials is still scarce.
• Challenges for sterilization, storage, and manufacture of BDDS have shown to be complex.
• Innovative drug-release biomaterials should be investigated case-by-case.
• The link between nanotechnology and drug-delivery properties provides wide opportunities for biomaterials development.

## Graphical abstract

**Figure:**
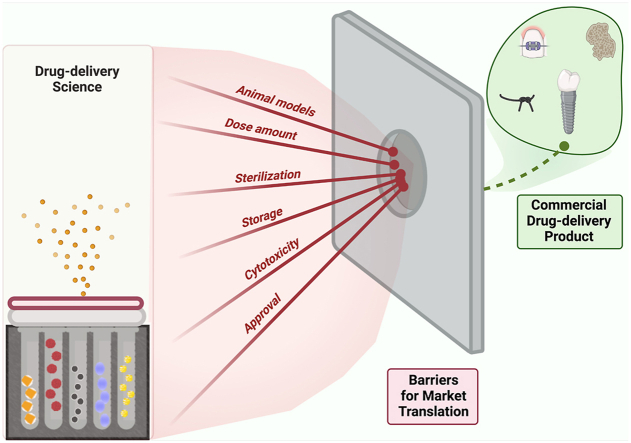

## 1 Introduction

The demand for rapid and predictable procedures and the requirement for specific treatments when patients seek dental treatments have promoted numerous basic investigations targeting biomaterials with innovative properties on the dental market [[1], [2], [3], [4]]. Among these materials, biomaterials containing drug delivery systems and nano-engineered structures have shown promising applications, such as those reported in oral rehabilitation with dental implants [5,6], guided bone regeneration applying bone substitutes and membranes [2,3,7], resins for dental reconstruction [8] and endodontic materials for the control of intracanal contamination [9].

In recent years, researchers have reported several alternatives for creating biomaterials for drug delivery systems (BDDS) and nano-engineered biomaterials; for example, adding nanoparticles to biomaterials, loading surfaces with pharmaceuticals or covering the biomaterial with bioactive coatings [[2], [3], [4], [5], [6], [7], [8], [9]]. However, almost none of these innovative methodologies have reached the dental market for clinical use. Firstly, the lack of availability is due to the complexity of manufacturing drug delivery biomaterials under current physical-chemical constraints. Secondly, basic commercial processes such as sterilization, the amount of drug incorporated and product validation are steps that require better investigation and clarification. Lastly, the storage method, product durability and market traceability are complex issues to be addressed.

Diverse literature can be found on preclinical studies addressing drug delivery systems on biomedical implant surfaces and nanotechnology [[4], [5], [6]]. However, only a small portion of these studies resulted in granted patents (25%), and a minimal portion (2%) became clinical products in the biomedical market [4]. Some studies demonstrated addition of different compounds/nanoparticles in membranes for bone regeneration with excellent biological and drug release results. However, it was unclear which sterilization process would be suitable for the new product and/or if the sterilization process could cause some damage to the biomaterials [[10], [11], [12]]. Moreover, the critical point of BDDS is drug maintenance until reaching the desired moment to apply it in preclinical models or clinical treatments. Few studies have reported strategies to successfully maintain the incorporated substance at the biomaterial safely over a long time, such as stabilizing the drug(s) with pH-responsive coatings or using processes to retain the molecules in the coating until their eventual intended application [13,14].

Lastly, the biggest challenges for the translation of BDDSs to the market are the clinical trials and their commercial approval by the responsible entities. Different reports have shown the difficulty of receiving commercial approval and/or demonstrate a major delay in the approval process [15,16] – consequences that often discourage funding bodies or even researchers themselves from continuing their development. Thus, the aim of this critical literature review is to clarify and detail the main steps and the main challenges involved in the development, analysis, and regulatory approval of BDDSs for clinical translation, as well as, the promising shift that biomaterials with drug-delivery systems and/or nano-engineered features may promote in the future clinical sector.

## 2 Methods to develop BDDSs for oral bone- and periodontal-related applications

### 2.1 Metal-based nanoparticles incorporation into biomaterials aiming for antibacterial properties and osteo-promotion

Nanoparticle incorporation has been reported as an important technique to create BDDSs, primarily with the use of metallic nanoparticles [17]. The application of metallic nanoparticles has been well explored in the literature using *in vitro* and *in vivo* models with a significant focus on antibacterial properties [[17], [18], [19], [20]]. Usually, metallic nanoparticles are added to the respective substrate or biomaterial by physical-chemical processes such as chemical synthesis, laser vaporization, spray-drying systems, sol-gel processes, photo-thermal reduction and catalytic chemical vapor deposition [17].

In dentistry, there are several applications of metal-based nanoparticle incorporation into biomaterials. The most relevant approaches related to bone and periodontal applications are demonstrated in periodontal procedures using antibacterial biomaterials for tissue regeneration [17] and drug delivery features for dental implant surfaces/bone substitutes/membranes [2,4,17,18]. Metallic nanoparticles have been associated with biomaterials such as hydroxyapatite, calcium fluoride, calcium phosphate, silica, chitosan and polymers in order to improve their features or their capacity for nanoparticle release [[17], [18], [19], [20], [21]]. Moreover, the shape, size and surface morphology of the nanoparticles were investigated as significant characteristics relating to BDDS effectiveness [21]. Heo et al. associated a biodegradable hydrogel with gold nanoparticles in order to create a “gelatin-like” material for bone tissue repair [22]. Their *in vitro* and *in vivo* findings showed improvement in proliferation, differentiation and expression of bone-related genes applying human adipose stem cells associated with the gelatin, furthermore, hydrogels loaded with different concentrations of gold nanoparticles promoted significant new bone formation in rabbit models [22]. In another report, Xu and co-authors added silver and strontium into porous scaffolds made by hydroxyapatite/chitosan aiming to improve bone regeneration and antibacterial properties. The antibacterial properties showed almost a total inhibition of *Staphylococcus aureus* (∼98%) and positive biocompatibility, osteoconductivity and mineralization, suggesting future application in tissue regeneration avoiding infections [23]. Additionally, drug release systems applying nanoparticles are normally associated with biodegradable coatings on biomaterials or with structures developed for the storage of nanoparticles on biomaterial surfaces [18,24].

Gunputh and colleagues showed the release of silver nanoparticles from TiO_2_ nanotubes with the objective of preventing contaminations on dental implants. The system demonstrated efficacy against *S. aureus*; however, no cytotoxicity tests were performed with bone-related/eukaryotic cells [25]. Nanostructured noble metal coating composed by palladium, gold and silver was added to implant surfaces in order to verify the osseointegration *in vivo*. The results showed anti-adhesive properties against *S. aureus* and osseointegration similar to standard implants without modern surface treatments [26]. One study has recently shown the synthesis of zirconium associated with calcium silicate for the development of a regenerative biomaterial; the authors tested Ca_3_ZrSi_2_O_9_ nanoparticles as bioceramic materials for bone substitute applications. The investigation demonstrated non-cytotoxic effects for these nanoparticles associated with bone marrow stem cells and completely bone regeneration after 6 weeks of healing in rabbit experimentation [27].

In addition, other authors revealed the application of gold nanoparticles that were incorporated into calcium phosphate cement for bone regeneration that resulted in a significant enhancement of osteogenic functions associated with dental pulp stem cells; furthermore, the study supported the slow release of the nanoparticles over 4 weeks [28].

### 2.2 Nanostructured surfaces and functionalization ability targeting molecule release

Firstly, implant surfaces associated with nanostructured topography have been currently reported as a significant strategy to reduce corrosion rates and generate higher biocompatibility [29], as well as, it has demonstrated mechano-antibacterial properties generating minimized taxes of bacterial adhesion [30]. Moreover, nanostructured surfaces have been demonstrated to be easily modified in terms of wettability, Kunrath and colleagues have demonstrated the hydrophilization of nanotextured surfaces by applying Ar/O_2_ reactive plasma [31] and Zhang and co-authors have shown the option of hydrophobization of nanotubular surfaces creating anti-fouling/antibacterial surfaces [32].

Techniques for surface loading using molecules have been intensively explored for dental implant surfaces [6,20]. Normally, the incorporation of compounds/molecules to surfaces requires specific sites where these compounds can be stored or loaded. In terms of dental implant surfaces, the development of nanoporous surfaces or nanotube surfaces has been investigated aiming drug/molecule storage until implant application in *in vitro* or *in vivo* models and thereafter in the clinical environment [33]. The most frequently investigated types of compounds are antibiotics, anti-inflammatory and pro-osteogenic substances, aiming to improve and protect the osseointegration process [6,20,33].

It has been demonstrated that vancomycin-releasing from nanotubular titanium surfaces expresses good antibacterial inhibition against *S. aureus* and potential quick drug release, suggesting future applications in dental implant components in order to prevent early infections [34]. Moreover, a report showed that TiO_2_ nanotubes loaded with antimicrobial peptides (AMPs) showing a release time of up to 7 days, resulted in an inhibition of 99% of *S. aureus* in *in vitro* models [35]. Sun et al. investigated loading of tetracycline on TiO_2_ nanotube surfaces, the study showed quick drug release (burst in 100 ​min); as a consequence, the surface antibacterial properties during this time period and the biocompatibility properties associated with *Porphyromonas gingivalis* and bone marrow stromal cells, respectively, were increased [36]. Several antibacterial compounds associated with implant surfaces have been investigated in recent years; as described above, nanotubular surfaces have been studied to incorporate metallic nanoparticles with the same final objective of controlling infections or contamination [18].

Additionally, compounds that may improve the bone regeneration process are the focus of research on surface loading. A study showed the development of hydrophilic nanotubular implant surfaces with BMP-2 (bone morphogenetic protein-2) incorporation [37]. The experimental results *in vitro* and *in vivo* showed better bone responses when compared to smooth surfaces without nanotubes, suggesting the significance of having specific sites for maintaining the compounds on the surface [37]. Icariin (ICA) was shown to be a promising osteoinductive compound for bone regeneration studies; some authors reported the application of ICA by simple physical absorption into TiO_2_ nanotube surfaces [38]. Releasing ICA improved the spreading, mineralization and expression of some bone-associated markers of bone marrow cells, suggesting a promising option for new implant surfaces [38].

Notably, the quantity of drugs or molecules loaded into nanoporous surfaces is largely dependent on nanopore geometry/size [6,39]. Some molecules may be larger than the space manufactured by porous surface development techniques, causing the deposition of these molecules on the surface and not specifically inside the pores. Reports have shown that the most interesting entrance measures for biological responses with nanoporous/nanotube surfaces are well below 150 ​nm [6,40,41]. For instance, delivery of Raloxifene (∼1 ​nm) and Alendronate (∼1.2 ​nm) has been demonstrated from 6 ​nm diameter pores on screw-shaped titanium implants [42]. Following this concept, molecules or compounds must be smaller than these dimensions to ensure optimal loading and subsequent release. Another issue promoted by this loading technique is uncontrolled rapid release. Since most surfaces cannot effectively control this parameter, burst release occurs as soon as the surface is exposed to a biological environment. Therefore, methods to control the release with coatings composed of different chemical biomaterials covering the nanostructured surfaces are currently being explored as the next step for drug delivery systems, leading to new possibilities for multifunctional release systems.

### 2.3 Application of multifunctional coatings (molecule immobilization for controlled release)

The application of coatings covering biomaterial surfaces has the objective to protect, amplify or/and improve the substrate properties. Usually, coatings are developed with synthetic or organic materials and may be associated with nanoparticles, drugs or other molecules to create multifunctional drug delivery systems [43]. The three main techniques used to design surfaces with coatings are physisorption or simple adsorption of the desired substance, immobilization by covalent binding and deposition of a *carrier* coating (e.g., polymers, hydrogels, and bioactive materials such as calcium phosphates, among others) [43,44]. Additionally, the material that is applied as a coating must have biocompatible properties when applied in biological environments. The surface morphology, roughness and wettability have an extreme influence on coating adhesion, while greater coating thickness may increase the total amount of compound that can be added to the carrier coating material [45].

In recent years, the development of calcium phosphate coatings has been explored for dental implants, suggesting better osseointegration [5,46,47] with osteoinductive potential [48]. However, achieving strong adhesion between the coating and the substrate surface for clinical applications can be challenging [49]. Alternatively, biodegradable coatings have been investigated aiming to be exposed to the *in vivo* environment with relative quick biodegradable properties [49], not promoting huge issues if coating ruptures occur *in vivo*. Implants with a calcium phosphate coating have been shown to improve resistance to push-out forces in both osteoporotic and healthy rats. Here, calcium phosphate coated implants led to a greater relative increase in mechanical anchorage in osteoporotic conditions compared to healthy rats, attributable to improvement the underlying osteopenia [46]. Alternatively, an amoxicillin-loaded polymeric coating was deposited covering anodized surfaces aimed for dental implantable components [50]. The authors demonstrated a positive dual-effectiveness for biocompatibility and antibacterial assays with bone-related cells and *S. aureus*/*S. epidermidis*, respectively, suggesting promising outcomes for polymeric coatings loaded with antibiotics in dental implant dentistry [50]. Moreover, the focuses of drug delivery systems and coatings application are not only explored for dental implants; researchers have been investigating their application for scaffolds or membranes for guided bone regeneration [[51], [52], [53], [54], [55]]. Applying antibiotics and PLGA/PCL nanofibers, one study demonstrated the development of nanofibers with sustainable-antibiotic release aiming at intra-pocket periodontal applications [55]. The authors suggested drug release lasting until 14 days without signals of toxicity in subcutaneous implantation using animal models, therefore, reporting as a beneficial material biocompatibility for periodontal applications [55]. Coatings associated with Osteostatin (peptide) were developed and deposited to porous-titanium scaffolds using a specific soaking technique [52]. The findings demonstrated the up-regulation of osteoprotegerin *in vitro* and increased bone regeneration using an *in vivo* model when Osteostatin was added to the scaffold compared to non-coated scaffolds [52]. Mathew et al. investigated a membrane for guided bone regeneration developed from medical grade polycaprolactone electrospun fibers plus azithromycin incorporated via solvent evaporation; the antibiotic release lasted for 14 days with strong antibacterial properties, suggesting a coating modification for alternative biomaterials employed in oral bone and periodontal regeneration [54].

**Fig. 1:**
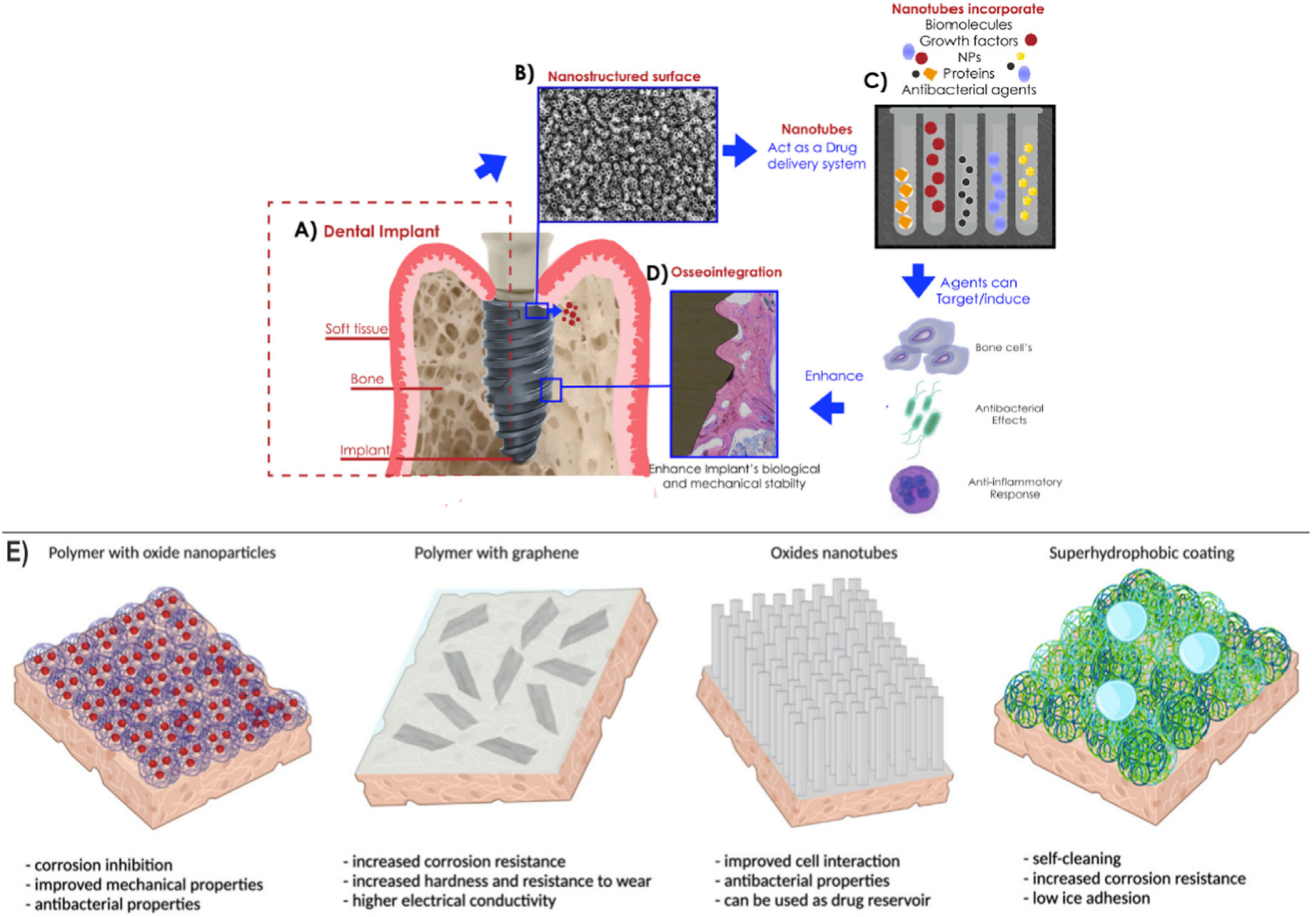
Scheme demonstrating the potential application of nanotubular surfaces as drug delivery systems and the possible benefits surrounding dental implants (A, B, C, and D). The nanotubular surfaces can be loaded with different drugs, molecules or peptides generating promising outcomes for bone-anchored metallic materials; adapted and reproduced with permission from Elsevier [4]. Different approaches applied to the development of multifunctional coatings aiming at drug loading and improved surface properties, such as corrosion resistance applying a polymeric layer loaded with nanoparticles, antibacterial features provided by nanotexturizations, and hydrophobicity, among others (E); reproduced with permission from Creative Commons Attributes (CC BY) [43].

From innovative methodologies using coatings with drug delivery properties, multifunctional surfaces, called “smart surfaces”, arise. Usually, their aim is to release more than one compound during their use or to have controlled properties in releasing drugs. As an example, Liu et al. developed a drug delivery system from a molecularly imprinted polymer that can be induced using NIR-light irradiation, promoting advanced methodologies for in deep tissue delivery [56]. Applying a different methodology, Dhavale et al. showed the development of a drug release coating pH-responsive; the authors proposed a system that stimulates compound release at acidic pHs, while at a neutral pH, the system remains relativity stable [57]. Likewise, drug delivery coatings are used to control the release of other compounds embedded within porous surfaces. A surface with TiO_2_ nanotubes loaded with interleukin-4 covered by an alginate/chitosan coating extended the release of this specific compound for 10 days [58]. Similarly, other authors revealed the possibility of loading composite scaffolds for bone regeneration with two or more drugs (anti-osteoporotic and anti-inflammatory drugs) using a tuning methodology creating a dual-drug co-delivery system [59], stimulating the bone healing process with multifunctional biomaterials, as shown in Fig. 1.

Innovative methodologies for creating BDDS have been reported in large numbers in recent years; however, almost all the new proposals are performed through *in vitro* or *in vivo* studies. Clinical studies or materials for oral rehabilitation with drug delivery systems are scarce in the current market due to the extreme difficulty in transferring technology to the market. There are many challenges to transferring a BDDS to the market, because it is considered a pharmacological biomaterial; therefore, considerations such as the amount of drug, sterilization, durability and regulations are of paramount importance and will be discussed in the following chapters.

## 3 Challenges

### 3.1 *In vitro* and preclinical models for evaluation of drug delivery biomaterials

After physical-chemical material development, innovative biomaterials should be investigated regarding biological responses and cellular mechanisms. The *in vitro* approaches applying different cell types are well established, easily controlled in clean environments and usually supported by significant previous literature. Several *in vitro* systems elucidated molecular pathways, the influence of biomaterial-surface properties and bacterial responses that are involved in the clinical application of innovative biomaterials, however, it is impossible to explore the complexity presented in organisms or in dynamic diseases related to the oral bone and periodontal tissue such as osteoporosis, diabetes, periodontitis, among others, using *in vitro* models [60]. For example, cell cultures involving multispecies or co-cultures may be applied to show detailed interaction between cells/substrates [60,61]; nevertheless, these methodologies can never totally reproduce the systemic responses of an animal or human [60].

In trying to overcome these challenges associated with *in vitro* methodologies, modern approaches have been tested in dental materials mimicking the oral tissues in 3D environments able to interact and mimic different cellular levels in function [62,63]. Furthermore, organs-on-a-chip have been reported as devices created to simulate organs and tissues physiology [62,[63], [64]]. These devices reproduce microenvironments and tissue architectures demonstrating promising properties to replicate tissue metabolism and organ function that are not possible by applying 2D *in vitro* experiments [62].

Investigations applying animal models are a significant step for successful product translation into the market. The *in vivo* experiments provide relevant outcomes by the use of multicellular organisms with their entire systemic system compared to *in vitro* single-cell studies. Complex bone- and periodontal-related healing processes are impossible to represent in *in vitro* models due to several mechanisms and influences caused by the multi-tissue organisms in different periods of time [60]. Therefore, for BDDS application, animal models are essential and must be well selected previously for experimental assays in order to demonstrate reliable results or to reveal negative responses prior to human tests. In order to demonstrate the advantages and disadvantages of each different preclinical methodology for application in innovative biomaterials research Table 1 is shown.

**Table 1:** Strengths and weaknesses of *in vitro* and preclinical approaches applied in current oral biomaterial studies.

Preclinical approaches	Main strengths	Main weaknesses
*In vitro* 2D models	- Easily approved; - Reproducibility; - Low experimental cost; - Controlled experimentation; - Fast results; - Supported by large literature; - Number of samples;	- Non-comparable to *in vivo* or clinical models; - Can not reproduce diseases; - Limited outcomes;
*In vitro* 3D models	- Allows to demonstrate some of the tissue responses; - Clean and controlled experimentation; - Simulate some of the tissue mechanisms; - Promote the reduction of animal experimentation;	- Not totally comparable to *in vivo* or clinical models; - Can not reproduce diseases; - Specific and planned outcomes;
Organs-on-a-chip	- Create microenvironments; - Simulate tissue- or organ- physiology; - Mimic specific tissues; - Clean experimentation; - Promote the reduction of animal experimentation;	- Not totally comparable to *in vivo* or clinical models; - Can not reproduce diseases; - Reduced literature; - Specific outcomes;
*In vivo* small mammals	- Reproducibility; - Supported by a large literature - Allows large-scale screening; - Faster healing times; - Cheaper than large animals; - Accelerated metabolism;	- Limited similarity to human organs and tissue environments; - Ethical approval needed; - Short life span;
*In vivo* large mammals	- Allows to simulate healing processes and periodontal conditions; - Extended healing times; - Allows to simulate oral tissues and oral environments with better similarity to humans; - Closer metabolism speed to humans;	- Highly expensive; - Complex ethical approval; - Number of samples; - Necessity of complex facilities for animal control;

However, animal models are not totally comparable to the human body; therefore, outcomes found in animals will normally reveal a perspective on possible human outcomes (see Table 2) [65,66]. Animal metabolism is a relevant matter for BDDS due to the necessity of investigating the drug release amount and the potential response caused by the released drug. Small animals such as rats possess short life spans (2–4 years); thus, the metabolism for drug absorbance or drug kinetics is accelerated, and the translation of these results to humans is not very reliable [65,66]. As a second issue with small animals, the requirement to create a BDDS with reduced size (small biomaterials) for application in the animal tissue will reduce the drug amount released compared to biomaterials manufactured for application in humans. Therefore, larger animals can provide more similar results to human metabolism and offer oral conditions with similarity in size to apply commercial dental drug-release biomaterials. Dogs, sheep, pigs and monkeys demonstrated life spans, weights and skeletal maturity levels not too distant from those of the human body [65,66], suggesting a significant impact for studies associated with drug-delivery biomaterials due to their physiological characteristics and metabolism not accelerated. Nevertheless, no animal model can be totally comparable to a human metabolism [60]; on the other hand, cost-effectiveness and sample numbers, ethical approval and facilities to care experimentation with large animals might be challenges that may support the application of smaller animals or *in vitro* studies. For that reason, these choices must be considered prior to starting any *in vivo* experimentation, and intense planning should be designed according to the main issues with animal experimentation, such as proximity to human physiology, bioethical implications, study timeline, animal gender, immunological features, nutrition aspects, and the number of animals [67]. Lastly, the conclusions derived from investigations with animal models should be described in detail, showing the possible limitations in each study, and demonstrating promising results that may be comparable to a future human study.

- *Potential solutions to overcome these challenges*:
  • Starting with *in vitro* experiments to show basic outcomes and advantages of using an intended novel biomaterial.
  • Conducting *in vivo* experiments in small animals (preferentially mammals) to provide a proof of concept.
  • Evolving the experimental work to large animals in order to compare the results with small animal models.
  • Evaluating the efficacy of biomaterials manufactured in sizes and/or drug loading comparable to the intended clinical applications.
  • Clinical translation should be pursued only after a successful outcome has been demonstrated in large animal models.

**Table 2:** Overview of animal physiological characteristics.

Animal characteristics	Mouse	Rat	Rabbit	Dog	Goat	Sheep	Pig	Human
Adult weight	12–30 ​g	70–300 ​g	1.5–2.5 ​kg	Beagle: 8.2–16 ​kg	45 ​kg avg	110 ​kg	50–350 ​kg	76–83 ​kg avg
Life span (years)	2	4	9	16	15	10–12	10	50–80
Age of skeletal maturity	Continuous growth	Continuous growth	10–11 months	12–18 months	3 years	15–18 months	12–14 months	16–18 years
Body temperature (°C)	36.5–37.3	37.5–39.5	38–39.5	38–39	38.3–39	38.5–39.5	38.3–38.8	36.5–37.5
Heart rate (beats per minute)	491–626	250–450	150–300	60–160	60–80	60–80	68–72	60–80

Table modified and adapted from Ribitsch et al. [65] and Muschler et al. [66].

Legends: "g" = grams; "kg" = kilograms; "avg" = average.

### 3.2 Dose of drug/molecule incorporated and released from biomaterials

As a first step for developing a BDDS is necessary to define the ideal amount of drug/compound to be incorporated into the substrate. The amount loaded depends on several factors, such as the biomaterial applied as substrate, coating thickness, incorporation process, and molecule size. Thus, most studies have measured the quantity of drug loading using advanced methodologies to weigh the samples previous/after loading and/or methodologies to calculate the cumulative drug release per time point [5,7,14,58,59]. Furthermore, the tissue responses created in biological environments are significant issues regarding drug delivery biomaterials. Usually, the drug release amount may generate benefit responses for some types of cells, but a totally opposite response for another specific cell. Several studies have demonstrated a dose-dependent response regarding the drug release surrounding different cells, showing that a high release amount can be cytotoxic to that same specific cell [57,68,69]. Tian et al. demonstrated a coating (strontium ranelate-loaded chitosan) that was deposited over titanium surfaces with drug release characteristics; the results showed positive responses in primary osteoblasts, such as enhanced proliferation, ALP activity and a higher expression of bone-associated markers at low concentrations (2–20 ​mmol/L); however, at higher concentrations (40–80 ​mmol/L), the same coating inhibited osteoblast growth [69]. Interestingly, another study showed that adding nanoparticulate zinc oxide to dental implant surfaces in different proportions resulted in non-significant differences for cytotoxicity tests (MTT and LDH) when using osteoblast-like cells (UMR-106 and MG-63) [70].

Another important issue about compounds incorporation into BDDS or nano-engineered biomaterials is the real amount that can be loaded onto the substrate. Normally, the amount of loading is presented in nano-doses, as demonstrated in studies applying nanotubes or nanoparticles [25,38,58]. The entire dose incorporated on the biomaterial surface might hardly reach to micro-dose due to the nano-scaled sites developed for loading. Moreover, the majority of studies on BDDS explored the drug release response by applying *in vitro* models and then tracking an interaction in the nano-micro-environment (nano-scaled drug release vs. cells) [38,39,69,70]. Differently, an *in vivo* study using dogs and rabbits evaluated the drug release from implants coated with doxycycline; the major release rate measured by the authors was 42 ​μg/mL after 72 ​h, and the results found non-significant differences in osseointegration parameters between coated and uncoated surfaces, suggesting a nonaggressive drug release system for the surrounding tissues [71]. However, the human body presents different velocities for drug degradation and kinetics compared to animal models [72], and clinical evaluations are of paramount importance to understand the correct compound amount to be loaded onto a clinical biomaterial.

Few reports have investigated the rates of drug release in patients due to the extreme difficulty of measuring the substances after insertion into the human body and/or the necessity of aggressive methodologies to analyze the surrounding tissues. Abtahi et al. inserted coated implants with a fibrinogen layer containing bisphosphonates in humans and reported an increased implant stability quotient after osseointegration (6 months); however, drug release evaluations were performed in an *in vitro* environment previous to implant applications [5]. In another study, dental implants with zoledronate coatings were tested in humans and analyzed in early healing periods (2–8 weeks), however, at this stage, no significance regarding osseointegration was found between coated and non-coated implants [73]. Additionally, the measurement of drug release in situ was not possible to be performed [73]. Biomaterials for bone regeneration with incorporated molecules such as BMP-2 were applied in patients with successful results; however, there was no analysis of the drug release rates after clinical application [74,75]. These reports show a lack of scientific description of the characterization of drug release biomaterials and few studies support methodologies to understand BDDSs when applied in a clinical environment, indicating the necessity of novel methods to analyze drug release after biomaterial application in humans.

- *Potential solutions to overcome these challenges*:
  • Physical and chemical characterization of BDDS previous to any *in vitro* or *in vivo* application.
  • Well-designed experiments to measure the amount of drugs/molecules loaded into the biomaterials. Advanced techniques to measure the rates of drugs/molecules released from biomaterials.
  • *In vitro* tests should address more than one concentration of drugs/molecules loaded in order to show possible cytotoxic effects on cells.
  • *In vivo* tests should corroborate outcomes found in *in vitro* studies and should verify the potential of drug/molecule degradation, migration and excretion.
  • Clinical tests must be supported for all the cited previous investigations and advanced techniques should be developed to analyze the drug release in patients.

### 3.3 Sterilization processes applied to nano-engineered biomaterials and BDDSs

**Fig. 2:**
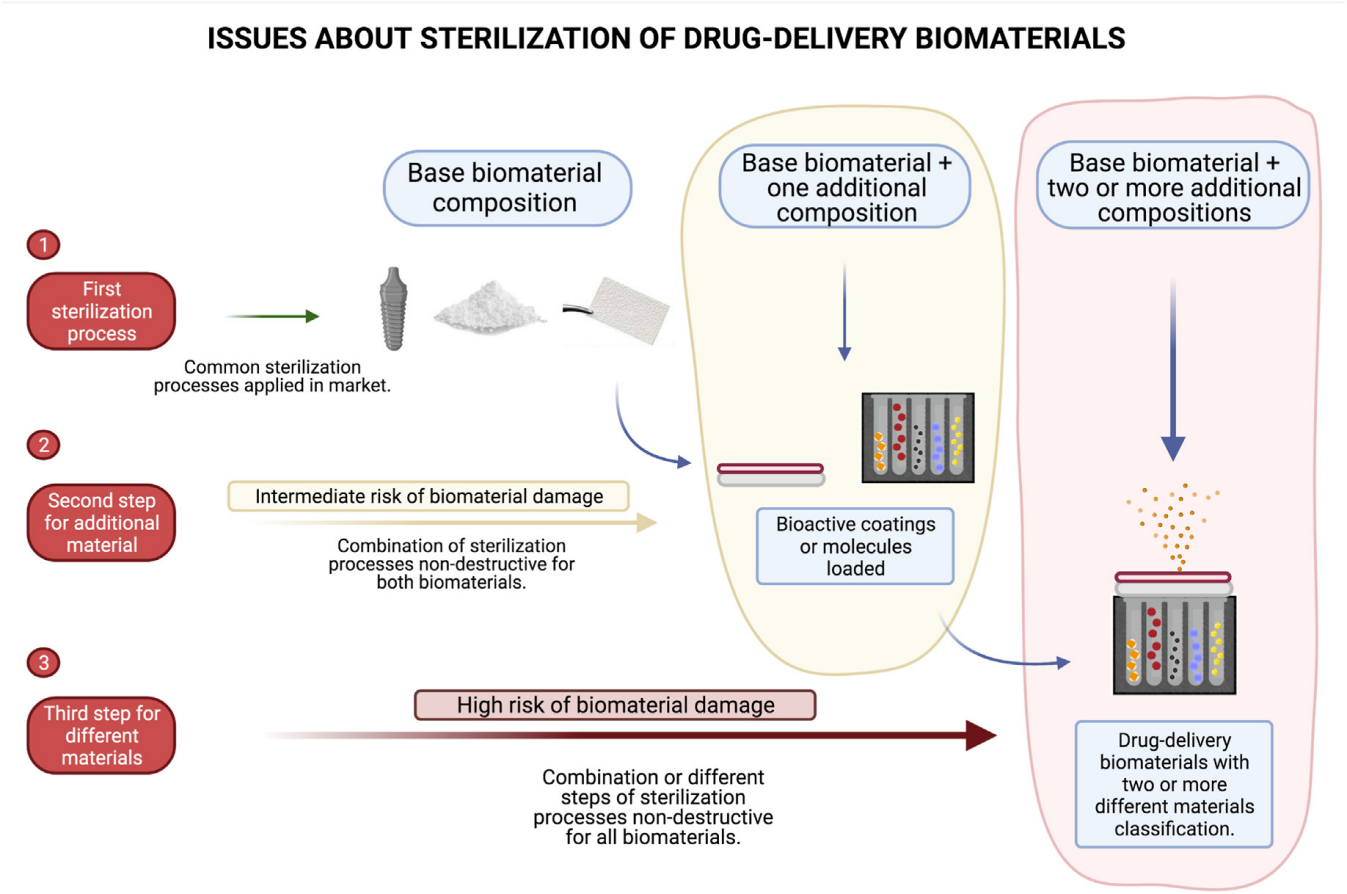
Scheme demonstrating the difficulties in promoting the complete sterilization of drug delivery biomaterials. Materials presenting different compositions are combined to create a single biomaterial; therefore, the processes applied for sterilization require safety for all the elemental compositions incorporated or added. 1- Common biomaterials without drug-delivery properties have standard protocols for sterilization. 2- Biomaterials loaded with molecules or coated with different chemical materials should have an approach that sterilizes both materials without damage. 3- Multifunctional biomaterials loaded with drugs and composed of different biocompatible materials must be sterilized with a controlled approach to not damage several chemical materials or apply multiple steps for sterilization.

Sterilization techniques are important at all study levels (*in vitro*, *ex vivo*, *in vivo* and clinical) and for the clinical application of biomaterials in patients. Sterilization processes may prevent possible contamination and misconstrue the results with cells, animal models and humans. Therefore, investigations proposing novel biomaterials or BDDS for application in biological environments require a significant description of the protocols applied for sterilization in study methodologies or patent reports. However, sterilization processes are slightly discussed in most of the studies published to date. Normally, the protocols for sterilization are reported in a few sentences and hardly ever, with a comparative analysis previous to/after the given sterilization process. This dearth of information explains one of the large gaps in translating innovative BDDSs to the clinical market and subsequently the appearance of BDDSs for application in patients. A novel biomaterial will not be approved by responsible agencies without a precise and safe protocol for complete material sterilization. The schematic in Fig. 2 represents the difficulty to create a sterilization protocol for BDDS involving more than one type of base biomaterial (e.g., metals ​+ ​drugs/metal ​+ ​molecules/metal ​+ ​polymers/metal ​+ ​polymers ​+ ​molecules, among others).

Metals have been very well investigated with respect to sterilization processes due to the numerous biomaterials present on the market today using these atomic compositions. Protocols using autoclaves, ultraviolet lights, ethylene oxide, reactive plasmas, and heating systems, among others, are effective in guaranteeing the successful application of metal devices in the clinical environment [76]. However, innovative drug delivery biomaterials have been associated with the addition of organic coatings or molecules that may be damaged by the effective sterilization processes used in metals. Baldin et al. reported the application of 3 different methods (steam autoclave, hydrogen peroxide plasma and ethylene oxide) for the sterilization of bioactive hybrid coatings containing hydroxyapatite particles; their study demonstrated negative consequences regarding the morphological structure and mechanical resistance for all the methods applied compared to non-sterilized samples [77]. Similarly, another study showed the application of 5 different methodologies for the sterilization of polymeric coatings, and they included autoclave, ultraviolet light, dry heat sterilization, ethylene oxide and gamma rays. The authors demonstrated that all these processes might cause some physical-chemical alterations in the studied materials, suggesting that the sterilization process must be investigated and tested for each material that is applied specifically [78]. Furthermore, gold nanoparticles were tested under different sterilization processes, showing that even metallic nanoparticles may have some physical-chemical alteration in their properties after using autoclaves, ultraviolet light (UV-light) and formaldehyde treatment [79]. Corroborating, a study has shown that the topography/structure of TiO_2_ nanotubes created by anodization for application in biomedical implant surfaces has been revealed to be compromised after autoclave sterilization due to the changes caused in the atomic structure by temperature and humidity [80].

These reports suggest that there is a complex decision before manufacturing BDDS regarding sterilization processes. Metallic biomaterials may support high temperatures, chemical processes and ionizing radiation with non-critical damage [81]. Natural and synthetic polymers have been reported to have a better performance after the ethylene oxide sterilization process [81]. In addition, biomaterials involving natural tissues or natural molecules require different methodologies, and the most promising technique is gamma irradiation due to non-critical damage to the biomaterial [81]. For BDDSs composed of two or more chemical compositions, applying a step-by-step sterilization process for each type of material during manufacture is suggested. This methodology might provide a reliable sterilization process and the possibility for the producers to verify the efficacy of the current process applied without compromising the previous chemical composition. The current literature is not totally clear to understand and to define flawless protocols for BDDS sterilization, and there is a substantial lack of scientific evidence to support the maintenance of the biomaterial physical-chemical properties after sterilization processes. For that purpose, a sterilization approach decided on a case-by-case basis with a consistent investigation should be designed for each study proposing BDDS. Table 3 shows the current suggested sterilization processes in the literature for different classes of chemical materials.

- *Potential solutions to overcome these challenges*:
  • Investigate separately all the chemical elements included in the composition of the novel biomaterials regarding the potential of damage with current sterilization processes.
  • Analyze different processes of sterilization previous to *in vitro*, *in vivo* or clinical studies, investigating the active maintenance of molecules/drugs without any damage.
  • Propose a step-by-step sterilization process when the novel biomaterial involves more than two or three different chemical classes of materials.

**Table 3:** The most common processes for biomaterials sterilization reported in the literature to materials presenting different chemical compositions and sterilization techniques reported in BDDS approaches.

Type of material	Sterilization processes suggested in the literature	Reference
Metals (alone)	Autoclave Heating systems UV- light Ethylene oxide Gamma irradiation Plasma application	[76,[79], [80], [81]]
Polymers	Ethylene oxide	[78,81]
Biological molecules	Ethylene oxide UV-light	[78,81]
Biological coatings	Ethylene oxide UV-light Gamma irradiation	[78,81]
Bone-particulate biomaterials	Ethylene oxide UV-light Gamma irradiation	[77,81]
Dental implants with bisphosphonate-coating	Gamma irradiation	[5]
Membranes loaded with Amoxicillin for dental applications	Not reported	[7]
Bacterial cellulose membranes loaded with chlorhexidine	Not reported	[11]
Nanotubular surface loaded with antimicrobial peptides	Ethanol (75%)	[13]
Coatings with hydroxyapatite nanoparticles loaded with antibiotics	Not reported	[14]
Porous scaffold loaded with Ag and Strontium	Not reported	[23]
Chitosan nanoparticle coatings on Titanium surfaces	UV-treatment (20 ​min per side)	[24]
TiO_2_ nanotubes loaded with antimicrobial peptides	Not reported	[35]
Titanium surfaces loaded with BMP-2	Not reported	[37]
TiO_2_ nanotubes loaded with Icariin	Not reported	[38]
PLGA-amoxicillin-loaded coatings on Ti surfaces	Low-temperature plasma of hydrogen peroxide	[50]
Nanoparticulate zinc oxide for orthopedic and dental implant coatings	Dipped in ethanol (100%) for 1 ​min and flamed.	[70]

### 3.4 Storage, packing and durability

Biomaterials that have been manufactured and sterilized correctly must be stored using a flawless technique for subsequent application in biological environments. For BDDSs, this process may require intense attention and innovative protocols to provide safety and durability for all the chemical compositions incorporated into the biomaterial. Some reports showed the maintenance of implant surface properties (hydrophilicity) employing techniques for storage under wet conditions [82,83], suggesting a protective environment until biomaterial application without the degradation of properties developed for benefic biological responses. Additionally, studies have demonstrated some approaches for applying polymeric coatings to protect the incorporated molecule/drug into the surface, inducing a sustainable release time due to the high degradation resistance of some polymers compared to the molecules added alone to the surface without protection [8]. Additionally, coated biomaterials with pH-responsive degradation have been reported, showing that the release and coating degradation occurred only under pH changes compared to neutral pH [84].

It is very well known that all types of materials are subject to some degradation process when exposed to the common oxygen due to the oxidation process, especially metals [85]. Most molecules, drugs and organic substances have a specific shelf life in the normal environment (when exposed to oxygen and environmental temperatures) and different shelf life under human body conditions (biological environment), which is normally called the “molecule half-life”, “drug half-life” or “shelf-life”. Therefore, BDDSs must be stored with protective capsules/coatings until their application; or their durability may be shorter than expected, creating another issue for market translation.

To provide an understanding of the durability of specific substances applied in BDDS studies, Table 4 shows some methodologies applied for the development of BDDSs, the most common substances discussed in the literature for creating drug delivery biomaterials, and details about the durability of these substances in different environments.

**Table 4:** Studies applying usual molecules to drug release systems, molecules’ shelf life details, and future perspectives to improve the durability of BDDS applying these specific molecules.

Substance/drug classification	Substance/Drug applied	Study references	Final application of BDDS	Drug release time (after application in study)	Substance/drug shelf-life characteristics in different environments	Future perspectives for extended durability (Recommendations)
Antibiotics	Amoxicillin	[7]	Nanocomposite membrane (electrospun nanocomsite matrices based on PCL ​+ ​nano-hydroxyapatite ​+ ​Amoxicillin).	21 days with effectiveness	Amoxicillin was most stable under pH values of 5–7 with a half-life of approximately 183 ​h. The pH and temperature are important factors for the degradability of this drug [86,87].	BDDSs applying amoxicillin require storage at the correct pH and may have improved durability if stored under frozen conditions.
		[55]	Nanofibers for periodontal diseases (PLGA and PCL nanofibers loaded with antibiotics by electrospinning).	14 days with effectiveness *in vivo*.		
		[50]	Dental implant surfaces (PLGA-Amoxicillin loaded coating covering Ti-anodized surface).	12 ​h with effectiveness in saliva medium.		
	Azithromycin	[54]	Membranes for guided bone regeneration (mPCL loaded with Azithromycin).	14 days with effectiveness *in vivo*.	The chemical stability of the antibiotic in aqueous systems remains unclear [88].	Processes applying encapsulation of the antibiotic into hydrogels or polymeric layers improved their stability [87].
Osteoinductors	Bisphosphonates	[89]	Coatings for biomaterials (biomimetic CaP layer formed by a sodium silicate process).	14 days with effectiveness *in vivo*.	The shelf life of Bisphosphonates is longer, it depends on the administration path or composition, and it may last for years in the human body or a normal environment [90].	Bisphosphonates have some advantages regarding storage due to their longer shelf life. The application of bisphosphonates in nanoparticulate compositions may lengthen the degradation time.
		[5]	Dental implant surfaces (cross-linked layer of fibrinogen loaded with bisphosphonates).	8 ​h with effectiveness *in vitro*.		
	BMP-2	[91]	Dental implant surfaces (biomimetic polyelectrolyte multilayer films loaded with BMP-2).	7 days with effectiveness *in vitro*.	The degradation of the entire BMP-2 molecule *in vivo* occurs in less than 2 weeks [91]. The integrity of coatings applied around the molecule is preserved for almost one year when stored at 4 ​°C [91]. At 37 ​°C, BMP-2 was digested quickly (in less than one week) and promoted microbial growth [92].	BMP-2 molecules require storage under cold conditions for extended durability. The incorporation of BMP-2 molecules into other carrier materials prolongs its shelf life.
		[92]	Dental implant surfaces (PLGA loaded with BMP-2 coating covering Ti-surfaces).	7 days with effectiveness *in vitro*.		
		[93]	Bone substitutes (three-dimensional porous scaffold based on mineralized recombinant human-like collagen loaded with BMP-2).	14 days with effectiveness *in vitro*.		
Anti-inflammatories	Ibuprofen	[94]	Bone substitutes (synthesis of hydroxyapatite nanostructured particles loaded with Ibuprofen).	24 ​h with effectiveness *in vitro*.	Ibuprofen showed accelerated degradation when exposed to different types of lights, with the best maintenance under dark conditions [96].	New biomaterials applying Ibuprofen are suggested to be manufactured with protective storage or coatings to maintain a dark environment.
		[95]	Implant surfaces (TiO2 nanotubes loaded with Ibuprofen).	12 ​h with effectiveness *in vitro*.		
	Diclofenac	[97]	Promising granulate bone substitutes (natural zeolite was loaded with Diclofenac by wet granulation).	8 ​h with effectiveness *in vitro*.	Diclofenac showed some unstable characteristics at pH 4 and 9 [98]. It was stable regarding degradation at 20 ​°C over 30 days [98].	Techniques for safe storage of drug release systems using diclofenac are necessary.

Common molecules/drugs, such as Amoxicillin, Ibuprofen, and BMP-2, among others, have been very well investigated in the literature due to their frequent applications in all areas of medicine. Most of these molecules/drugs present faster degradation rates in biological environments and in natural environments (25 ​°C temperature) [[86], [87], [88], [89], [90], [91], [92], [93], [94], [95], [96], [97], [98]] compared to colder environments. However, when stored under high cold conditions or with protective coatings, they demonstrated extended shelf lives [86,87,91]. In the dentistry market, drug delivery biomaterials are rare; therefore, details of the appropriate storage protocol for this class of biomaterial remain unclear in the literature.

Another unclear issue about BDDSs is related to their transportation and storage in capsules to protect the drug delivery systems associated with the biomaterials. Some specific materials used to store the biomaterials might interact with or contaminate the surface. Investigations revealed the appearance of impurities attached to the surface of implantable devices that may be related to the fabrication or storage processes [99,100]. Moreover, the method used to transport BDDS after sterilization and storage should be analyzed. The constant movements caused by transportation from the company/laboratory to market/clinical locals may influence the durability or may generate the activation of the drug delivery system, as well as, the temperature conditions associated with the transport should be verified. Solutions for producing innovative BDDSs must be explored case-by-case with relevant analysis to report the durability of each compound incorporated into the biomaterial before submission for market agency approval.

- *Potential solutions to overcome these challenges*:
  • “Shelf life” investigation of molecules/drugs added to the biomaterials.
  • To investigate the alternatives for extended “shelf life” using storage in wet solutions, low temperatures and customized holders to preserve integrity during transportation.
  • To explore alternatives to protect the incorporated molecules/drugs with different resistant coatings when applied in tough biological environments.

## 4 Counterpoints for drug release systems in biomaterials

Innovative biomaterials promote technological evolution in medical science and are usually focused on benefits for patients; however, these technological advances may have negative reactions in some cases, primarily when applying drug delivery biomaterials localized to singular tissues without adequate investigation.

In the first instance, the application of bioactive coatings covering biomaterial substrates may severely influence topographical properties aimed at faster healing. Kazek-Kęsik and co-authors applied a polymeric coating loaded with antibiotic covering an anodized surface and revealed severely changes in roughness properties, as well as, alteration in wettability changing the surface from a hydrophilic surface to a hydrophobic surface [50]. Additionally, surface nanostructuration methodologies have been shown to modify the crystalline phase and mechanical properties of Titanium and Ti-alloys [101]. Mechanical stability and appropriate protocols to develop these innovative surfaces are important in order to not create any delamination or breakage during biomaterial insertion promoting the release of fragments that might initiate inflammatory/cytotoxic responses [101].

**Fig. 3:**
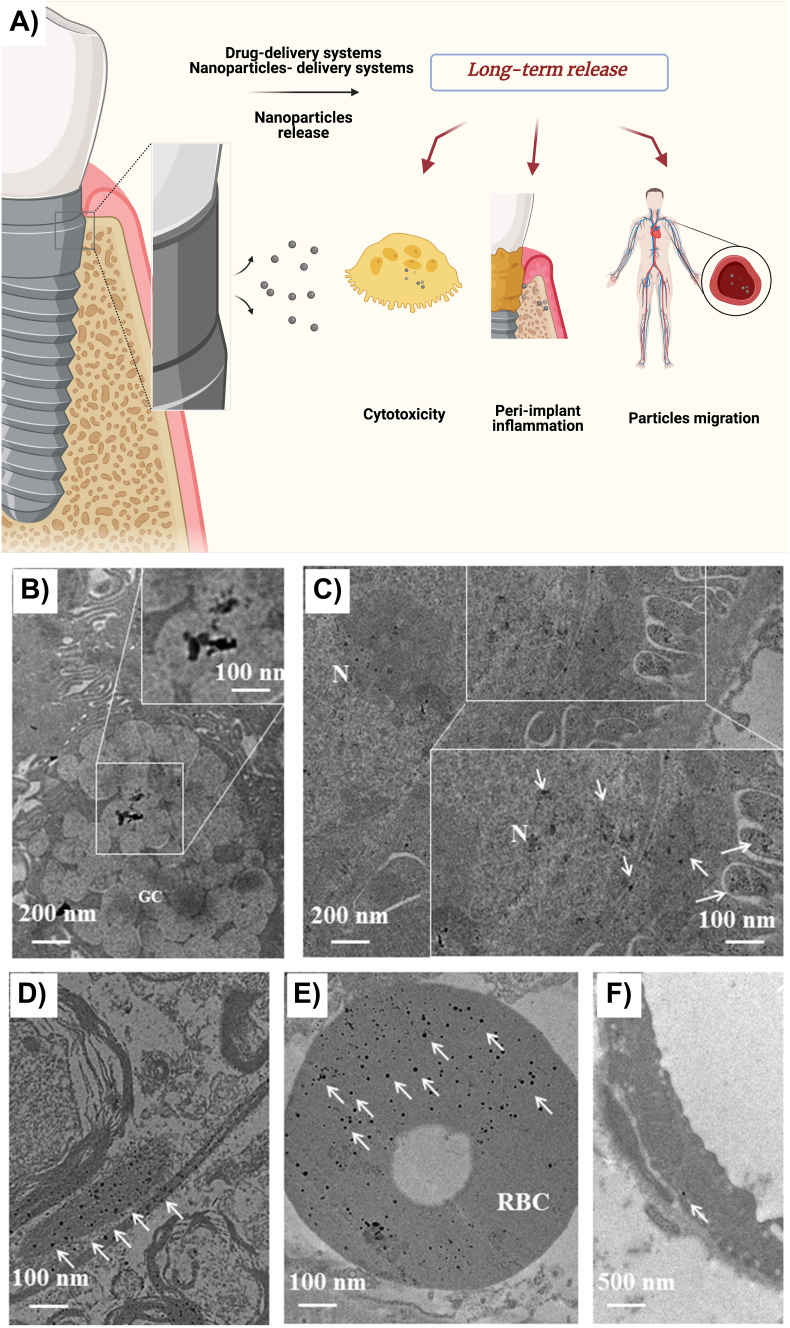
Scheme demonstrating the possible routes for metallic nanoparticles released from long-term exposure in the human body (A). Metallic nanoparticles found in murine models after biodistribution via blood circulation (B, C, D, E, and F). White arrows indicate Ag nanoparticles in the intestine (B), kidney (C), brain (D), red blood cells (E) and endothelial cells (F). Images adapted and reproduced with permission from Creative Commons Attributes (CC BY) [103].

Another of the possible adverse effects related to BDDSs may be cytotoxicity caused by molecules/nanoparticles release around the biomaterial; studies have shown that excessive drug exposure induces cytotoxicity responses in different tissues [57,68,69]. Moreover, the same statement can be used for the application of metallic nanoparticles; the release of gold nanoparticles from modified surfaces demonstrated dose-dependent cytotoxicity when they were released in higher amounts together with specific cells (NIH-3T3 fibroblasts and 4T1 tumor cell lines) [102]. Additionally, silver nanoparticles inserted in murine models have been found in several tissues distant from the surgical site, indicating the possible migration, transportation and biodistribution of these nanoparticles around the circulation and cells due to the nano-scaled size (Fig. 3) [103]. Studies have shown that the presence of metallic nanoparticles or ions around implantable devices may impact the progress of peri-implant diseases in the long term exposition due to foreign body reactions, DNA methylation, or changes in the oral microbiome [104].

The bioaccumulation of specific metals in the human body is a critical issue for metallic devices due to the potential to create allergies or autoimmune responses; however, the literature still not reveals a consensus about which type of implantable devices may critically induce these problems [18,105]. Therefore, studies and innovative products using drug release systems must provide substantial information about possible cytotoxicity effects and the probable excretion/degradation pathway of the drug that was released.

A second important issue is the creation of localized antibiotic resistance due to the incorporation of antibiotics, antimicrobials, or peptides derived from antibiotics, into the BDDS. There is an increased demand worldwide to restrict the inappropriate use of antibiotics to control drug resistance [106]. The application of BDDS with antibiotics must demonstrate safety, effectiveness and a flawless drug release amount over a controlled time. Biomaterials releasing small doses of antibiotics with quick burst times must be tested against resistant bacteria, providing reliable ways to evolve for market products. Or alternatively, these innovative products may stimulate the evolution of antibiotic-resistant bacteria.

- *Potential solutions to overcome these challenges*:
  • Broad characterization applied to *in vitro* experiments and different concentrations of drugs/molecules loaded.
  • Evaluation of the advantages to include drug-release biomaterials compared to the biomaterials already present in the market.

## 5 “Bench-to-bedside” – significant considerations for translating preclinical scientific evidence into market products

**Fig. 4:**
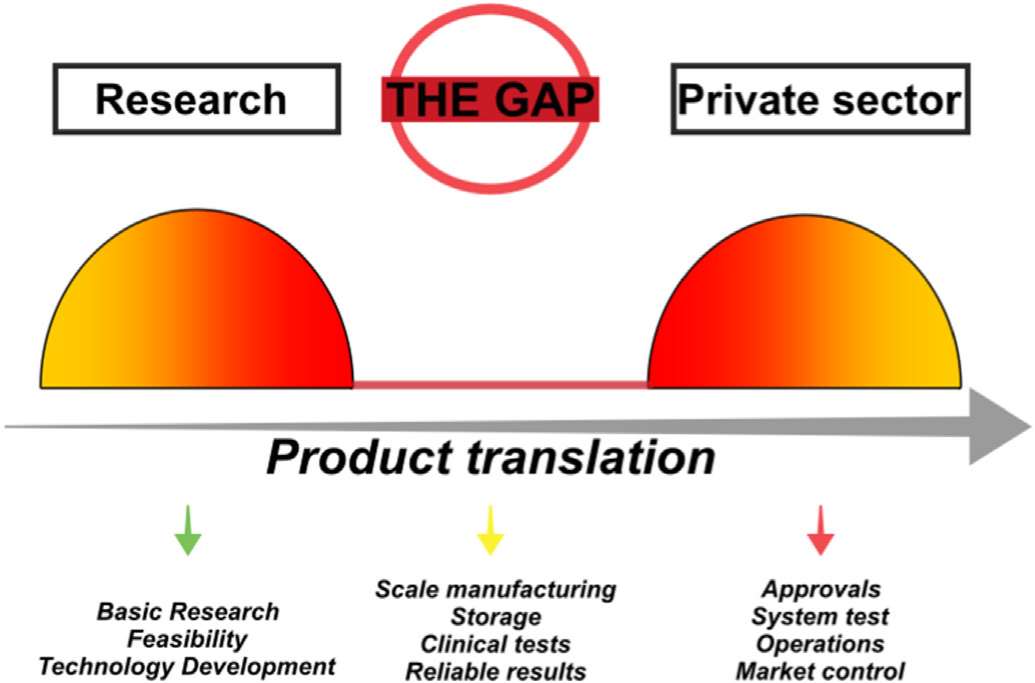
Scheme showing the main issues involved in translating basic research on BDDS to the private sector. The “gap” is created due to the broad and tough level of requirements prior to market approval, requiring several years of research delaying the release of new products onto the market.

The final step in translating an innovative drug delivery product for the market application is related to receiving approval from the controlling agencies and, in an appropriate order, the release into the market. However, this step has been reported as “the valley of death” due to the complexity of completing the product translation [16,107]. Three main points have been established regarding the requirements for successful translation and to overcome this gap: 1) basic research focused on translational products; 2) technology validation for subsequent company development; and 3) company formation with a focus on large-scale production [16] (Fig. 4).

Having a complete product project, the manufacturers in order to receive biomaterial validation require heedfulness regarding future perspectives on the market, such as safety, efficiency and customer expectations. The innovative product must have previous studies and tests for safety and efficacy to be submitted for approval. Moreover, the future expectations of clients that will use/acquire the product must be highly promising, or the new product might be undersold.

The main applications of drug delivery systems for biomaterials have been focused on specific treatments using the localized release of some drugs, molecules or particles with the aim of being highly specialized [108,109]. The reason behind this specialization is the improvement of a specific treatment for patients compromised with a given disease or deficient health condition. In fact, some drug delivery biomaterials have been investigated with the objective of being translated as a universal product in the market to reach all common patients independent of health conditions. It has been reported that ∼18 million implants are sold annually by different companies [110]. Despite the high rate of successful osseointegration [110], dental implants and biomaterials related to tissue regeneration still show early and late complications [[111], [112],^113^] and it promotes the need for innovation, moreover, the dental market has been reported to be growing quickly, showing a prediction of USD 1.3 billion invested on implant/peri-implantitis treatments and over USD 5.7 billions invested on oral treatments involving biomaterials [114,115].

Nevertheless, BDDSs require specialized analyses of the future market that it will be launched in order to understand and recognize the range of promising clients; the requirement for this technology in the market; and if the cost-effectiveness will be accessible for the common clients. Some biomaterials without drug delivery properties have shown excellent results on the current market and are commonly used by the general public; in addition, the elevated cost caused by the production of a BDDS may lead to a product underselling if it does not demonstrate a substantial improvement in the dental market. For example, the application of BDDS in cancer therapy was valued at USD 4.31 billions in 2016 and continues to increase [116], due to intense demand to promote better and more effective therapies for patients needing cancer treatments [[116], [117], [118]]. Therefore, the investments in technology and market translation are faster and have high justification.

Additionally, it is very important to comprehend where (countries and continents) the product is aimed at the market. BDDSs are considered materials with pharmacological properties because of their capacity to release drugs or maintain molecules loaded until their use [18]. Health agencies such as the European Medicines Agency (EMA), FDA (US Food and Drug Administration) and Anvisa (Brazilian Health Agency) classify this type of biomaterial as a “combined device”; therefore, the requirements for approval are critically elevated [18,[119], [120], [121], [122]].

Moreover, some minor differences between these requirements have been consistently revealed by different agencies around the world, since some products can be approved in one country and the same product cannot be released on another continent. As a classic example, dipyrone is permitted in some countries with unblocked consumption; however, the FDA in the U.S. and its counterparts in several countries prohibited its common use [123]. Furthermore, the time for approval differs between agencies around the entire world. Lastly, critical analyses of the rules/laws around which country (ies) is/are want to release an innovative biomaterial are necessary and may determine its success during the application for approval.

After successful approval by the health agencies, another phase starts in relation to biomaterial control on the market. Since it is considered a “drug device”, the necessity of having full traceability from the company production until the application to a patient is essential. Therefore, BDDSs must not remain stored for long periods of time before being sold or applied to patients, due to the presence of pharmacological compounds and exclusive shelf life. For that reason, the after-development control will generate expensive costs for the company to manage the commercialization process perfectly until clinical application.

## 6 Future perspectives

### 6.1 Future perspectives on clinical impact of dental drug-delivery biomaterials

As described in the last chapters, drug-delivery biomaterials for dental implants and periodontal applications still are not widely present in the common market. However, the large quantity of basic and preclinical studies has shown a great range of possibilities for advances on future clinical treatments compared to biomaterials currently applied in oral rehabilitation. The aim for clinical impact observed in the literature, focus on the improvement of specific treatments in oral regeneration such as osseointegration of implants, accelerated bone regeneration, infection control and improvement of bone quality [4,124,125]. Drug-delivery systems associated with biomaterials target one localized site by applying their property of drug release in a concentrated way, therefore, increasing the beneficial responses in the desired site. Moreover, the application of one or two specific molecules/nanoparticles in localized regions in which the tissues may be compromised might decrease largely the use of systemic drugs that normally spread to the entire human organs [20,126].

The application of these biomaterials in patients presenting systemic diseases such as diabetes, osteoporosis, bone-related deficiencies, immunological diseases or in order to combat/prevent bacterial infections may generate future better outcomes for treatments using oral biomaterials and dental implants than the rate of success reached currently. Measurements about the clinical impact of BDDSs still are not evident in the literature in order to be reported here, however, overpassing the challenges for manufacturation and market approval, BDDSs shown promising abilities to advance personalized oral treatments removing the large application of pharmacological drugs using systemic administration.

### 6.2 Reduction of systemic administration of antibiotics

Significantly, BDDSs have been intensely explored in order to control infections and the adhesion of bacteria on the substrates applied [11,20,127,128]. Several techniques have shown possibilities to incorporate antibiotics, peptides, or antibacterial molecules into oral biomaterials [11,20,127,128]. The main focus has been shown in order to deliver these drugs/molecules directly to the targeted site where the compound should act with significant performance during some period of time [129].

Therefore, this property developed in these multifunctional biomaterials may improve one of the biggest issues in the current worldwide healthcare system related to antibiotic resistance in patients [106,130]. The reduction of systemically antibiotics administration decreases the development of new bacterial resistant species, improving the entire ecosystem involved in oral procedures and in general healthcare, generating higher taxes of success. Research has demonstrated the reduction of bacterial adhesion and proliferation by applying BDDSs associated with antibacterial compounds in the early stages of biomaterial application, showing the fundamental action to combat early infections and suggesting a reduction of the use of systemic antibiotics [50,55]. However, late infections still might be tough to control due to the commonly quick release of the molecules from BDDSs lasting approximately from hours to one month with safety in the best technologies [55,130]. Further studies are still necessary to testify the efficacy of antibiotic-loaded biomaterials in order to remove the application of systemic antibiotics; meanwhile, this particular property may be a future “change of paradigms” in the science-related to microorganisms.

## 7 Concluding remarks

BDDSs are a promising group of biomedical devices that will allow for broadening the patient inclusion criteria. For instance, patients with certain health conditions that are experiencing unsuccessful treatments in oral regeneration. Therefore, acceleration of the healing process and the rise of antibacterial properties, associated with the development of personalized dental biomaterials, have been demonstrated to be the main focus of drug delivery biomaterials for oral tissue applications [4,[131], [132], [133]]. The translation of all current basic science into market products has a possibility for substantial improvements in oral treatments and general medicine. Thus, the potential of decreasing waiting time for oral regeneration treatments, improving tissue regeneration in patients with compromised health conditions, or controlling bacterial infection around biomaterials inserted into the oral environment, solves most of the problems related to unsuccessful biomaterial applications reported in the literature [[134], [135], [136], [137]]. Additionally, the scientific content explored in this review may be translated to all bone-anchored biomaterials aiming at drug release for applications in the human body, such as orthopedic implants, spinal implants, bone substitutes, craniofacial devices, membranes for bone regeneration, cancer therapy, among others. The technology for drug delivery biomaterials can be applied to a wide range of biomaterial surfaces composed of different substrates and can be modified for specific tissue applications.

However, the huge gap between investigations performed in laboratory stages and products available in the market needs to be minimized in the future. The concept of developing a drug delivery biomaterial with a specific additional compound creates the necessity to explore all processes and reactions associated with this particular chemical composition, analyzing the entire manufacturing process, storage stage and after-selling traceability periods applying a unique approach for this innovative system.

Currently, from our knowledge, no nano-engineered BDDSs applied to oral bone or periodontal regeneration are released on the market and substantial information about the advantages of these systems compared to current products available is unclear to state to date. Future studies providing clinical comparisons between biomaterials with drug release systems and non-functionalized biomaterials will show the possible advantages of these advances. Notably, some issues reported in the current literature and revealed in clinical situations regarding the application of biomaterials related to oral bone and periodontal tissue regeneration with unsuccessful treatments remain constant and without appropriate remedy [[111], [112],^113^]; thus, BDDSs may provide a pathway for solving the problems reported to date and ultimately find their proper place in the market. Finally, a new generation of biomaterials with drug delivery systems will appear on the market in the near future, promoting optimal and localized treatments that are not present in the current range of treatments.

## Declaration of competing interest

The authors declare the following financial interests/personal relationships which may be considered as potential competing interests:Marcel F. Kunrath reports financial support was provided by 10.13039/501100007619Osteology Foundation.

## Data Availability

No data was used for the research described in the article.

## References

[1] Omar O., Elgali I., Dahlin C., Thomsen P. (2019). Barrier membranes: more than the barrier effect?. J. Clin. Periodontol..

[2] Eivazzadeh-Keihan R., Bahojb Noruzi E., Khanmohammadi Chenab K., Jafari A., Radinekiyan F., Hashemi S.M. (2020). Metal-based nanoparticles for bone tissue engineering. J. Tissue Eng. Regener. Medic..

[3] Donos N., Dereka X., Calciolari E. (2019). The use of bioactive factors to enhance bone regeneration: a narrative review. J. Clin. Periodontol..

[4] Kunrath M.F., Diz F.M., Magini R., Galárraga-Vinueza M.E. (2020). Nanointeraction: the profound influence of nanostructured and nano-drug delivery biomedical implant surfaces on cell behavior. Adv. Colloid Interface Sci..

[5] Abtahi J., Tengvall P., Aspenberg P. (2012). A bisphosphonate-coating improves the fixation of metal implants in human bone. A randomized trial of dental implants. Bone.

[6] Kunrath M.F., Hubler R., Shinkai R.S., Teixeira E.R. (2018). Application of TiO2 nanotubes as a drug delivery system for biomedical implants: a critical overview. ChemistrySelect.

[7] Furtos G., Rivero G., Rapuntean S., Abraham G.A. (2017). Amoxicillin-loaded electrospun nanocomposite membranes for dental applications. J. Biomed. Mater. Res. Part B: Appl Biomater.

[8] Imazato S., Kitagawa H., Tsuboi R., Kitagawa R., Thongthai P., Sasaki J.I. (2017). Non-biodegradable polymer particles for drug delivery: a new technology for “bio-active” restorative materials. Dent. Mater. J..

[9] Cuppini M., Zatta K.C., Mestieri L.B., Grecca F.S., Leitune V.C.B., Guterres S.S., Collares F.M. (2019). Antimicrobial and anti-inflammatory drug-delivery systems at endodontic reparative material: synthesis and characterization. Dent. Mater..

[10] Shanmugasundar S., Kannan N., Sundaravadivel E., Zsolt S., Mukunthan K.S., Manokaran J. (2019). Study on the inflammatory response of PMMA/polystyrene/silica nanocomposite membranes for drug delivery and dental applications. PLoS One.

[11] Inoue B.S., Streit S., dos Santos Schneider A.L., Meier M.M. (2020). Bioactive bacterial cellulose membrane with prolonged release of chlorhexidine for dental medical application. Int. J. Biol. Macromol..

[12] Hasani-Sadrabadi M.M., Sarrion P., Nakatsuka N., Young T.D., Taghdiri N., Ansari S. (2019). Hierarchically patterned polydopamine-containing membranes for periodontal tissue engineering. ACS Nano.

[13] Chen J., Shi X., Zhu Y., Chen Y., Gao M., Gao H. (2020). On-demand storage and release of antimicrobial peptides using Pandora's box-like nanotubes gated with a bacterial infection-responsive polymer. Theranostics.

[14] Geuli O., Metoki N., Zada T., Reches M., Eliaz N., Mandler D. (2017). Synthesis, coating, and drug-release of hydroxyapatite nanoparticles loaded with antibiotics. J. Mater. Chem. B.

[15] Linton J.D., Xu W. (2021). Understanding and managing the biotechnology valley of death. Trends Biotechnol..

[16] Taylor D.P., Yoshida M., Fuller K., Giannobile W.V., Sfeir C.S., Wagner W.R., Kohn D.H. (2021). Translating dental, oral, and craniofacial regenerative medicine innovations to the clinic through interdisciplinary commercial translation architecture. J. Dent. Res..

[17] Bapat R.A., Joshi C.P., Bapat P., Chaubal T.V., Pandurangappa R., Jnanendrappa N. (2019). The use of nanoparticles as biomaterials in dentistry. Drug Discov. Today.

[18] Kunrath M.F., Campos M.M. (2021). Metallic-nanoparticle release systems for biomedical implant surfaces: effectiveness and safety. Nanotoxicology.

[19] Song W., Ge S. (2019). Application of antimicrobial nanoparticles in dentistry. Molecules.

[20] Kunrath M.F., Leal B.F., Hubler R., de Oliveira S.D., Teixeira E.R. (2019). Antibacterial potential associated with drug-delivery built TiO2 nanotubes in biomedical implants. Amb. Express.

[21] Jandt K.D., Watts D.C. (2020). Nanotechnology in dentistry: present and future perspectives on dental nanomaterials. Dent. Mater..

[22] Heo D.N., Ko W.K., Bae M.S., Lee J.B., Lee D.W., Byun W. (2014). Enhanced bone regeneration with a gold nanoparticle–hydrogel complex. J. Mater. Chem. B.

[23] Xu Z.L., Lei Y., Yin W.J., Chen Y.X., Ke Q.F., Guo Y.P., Zhang C.Q. (2016). Enhanced antibacterial activity and osteoinductivity of Ag-loaded strontium hydroxyapatite/chitosan porous scaffolds for bone tissue engineering. J. Mater. Chem. B.

[24] Poth N., Seiffart V., Gross G., Menzel H., Dempwolf W. (2015). Biodegradable chitosan nanoparticle coatings on titanium for the delivery of BMP-2. Biomolecules.

[25] Gunputh U.F., Le H., Handy R.D., Tredwin C. (2018). Anodised TiO2 nanotubes as a scaffold for antibacterial silver nanoparticles on titanium implants. Mater. Sci. Eng. C.

[26] Svensson S., Suska F., Emanuelsson L., Palmquist A., Norlindh B., Trobos M. (2013). Osseointegration of titanium with an antimicrobial nanostructured noble metal coating. Nanomed. Nanotechnol. Biol. Med..

[27] Doostmohammadi A., Karimzadeh Esfahani Z., Ardeshirylajimi A., Rahmati Dehkordi Z. (2019). Zirconium modified calcium-silicate-based nanoceramics: an in vivo evaluation in a rabbit tibial defect model. Int. J. Appl. Ceram. Technol..

[28] Xia Y., Chen H., Zhang F., Bao C., Weir M.D., Reynolds M.A. (2018). Gold nanoparticles in injectable calcium phosphate cement enhance osteogenic differentiation of human dental pulp stem cells. Nanomed. Nanotechnol. Biol. Med..

[29] Liu Z., Liu Y., Liu S., Wang D., Jin J., Sun L. (2021). The effects of TiO2 nanotubes on the biocompatibility of 3D printed Cu-bearing TC4 alloy. Mater. Des..

[30] Li H., Cui Q., Feng B., Wang J., Lu X., Weng J. (2013). Antibacterial activity of TiO2 nanotubes: influence of crystal phase, morphology and Ag deposition. Appl. Surf. Sci..

[31] Kunrath M.F., Correia A., Teixeira E.R., Hubler R., Dahlin C. (2022). Superhydrophilic nanotextured surfaces for dental implants: influence of early Saliva contamination and wet storage. Nanomaterials.

[32] Zhang L., Zhang L., Yang Y., Zhang W., Lv H., Yang F. (2016). Inhibitory effect of super-hydrophobicity on silver release and antibacterial properties of super-hydrophobic Ag/TiO2 nanotubes. J. Biomed. Mater. Res. Part B: Appl Biomater.

[33] Gulati K., Ivanovski S. (2017). Dental implants modified with drug releasing titania nanotubes: therapeutic potential and developmental challenges. Expet Opin. Drug Deliv..

[34] Ionita D., Bajenaru-Georgescu D., Totea G., Mazare A., Schmuki P., Demetrescu I. (2017). Activity of vancomycin release from bioinspired coatings of hydroxyapatite or TiO2 nanotubes. Int. J. Pharm. (Amst.).

[35] Ma M., Kazemzadeh-Narbat M., Hui Y., Lu S., Ding C., Chen D.D. (2012). Local delivery of antimicrobial peptides using self-organized TiO2 nanotube arrays for peri-implant infections. J. Biomed. Mater. Res., Part A.

[36] Sun L., Xu J., Sun Z., Zheng F., Liu C., Wang C. (2018). Decreased porphyromonas gingivalis adhesion and improved biocompatibility on tetracycline-loaded TiO2 nanotubes: an in vitro study. Int. J. Nanomed..

[37] Li Y., Song Y., Ma A., Li C. (2019). Surface immobilization of TiO2 nanotubes with bone morphogenetic protein-2 synergistically enhances initial preosteoblast adhesion and osseointegration. BioMed Res. Int..

[38] Zhang Y., Liu C., Chen L., Chen A., Feng X., Shao L. (2018). Icariin-Loaded TiO2 nanotubes for regulation of the bioactivity of bone marrow cells. J. Nanomater..

[39] Çalışkan N., Bayram C., Erdal E., Karahaliloğlu Z., Denkbaş E.B. (2014). Titania nanotubes with adjustable dimensions for drug reservoir sites and enhanced cell adhesion. Mater. Sci. Eng. C.

[40] Gong Z., Hu Y., Gao F., Quan L., Liu T., Gong T., Pan C. (2019). Effects of diameters and crystals of titanium dioxide nanotube arrays on blood compatibility and endothelial cell behaviors. Colloids Surf. B Biointerfaces.

[41] Wang Y., Wen C., Hodgson P., Li Y. (2014). Biocompatibility of TiO2 nanotubes with different topographies. J. Biomed. Mater. Res., Part A.

[42] Harmankaya N., Karlsson J., Palmquist A., Halvarsson M., Igawa K., Andersson M., Tengvall P. (2013). Raloxifene and alendronate containing thin mesoporous titanium oxide films improve implant fixation to bone. Acta Biomater..

[43] Zafar M.S., Fareed M.A., Riaz S., Latif M., Habib S.R., Khurshid Z. (2020). Customized therapeutic surface coatings for dental implants. Coatings.

[44] Olmo J.A.D., Ruiz-Rubio L., Pérez-Alvarez L., Sáez-Martínez V., Vilas-Vilela J.L. (2020). Antibacterial coatings for improving the performance of biomaterials. Coatings.

[45] Alkekhia D., Hammond P.T., Shukla A. (2020). Layer-by-layer biomaterials for drug delivery. Annu. Rev. Biomed. Eng..

[46] Alghamdi H.S., Cuijpers V.M.J.I., Wolke J.G.C., Van den Beucken J.J.J.P., Jansen J.A. (2013). Calcium-phosphate-coated oral implants promote osseointegration in osteoporosis. J. Dent. Res..

[47] Tsui Y.C., Doyle C., Clyne T.W. (1998). Plasma sprayed hydroxyapatite coatings on titanium substrates Part 2: optimisation of coating properties. Biomaterials.

[48] Ripamonti U., Roden L.C., Renton L.F. (2012). Osteoinductive hydroxyapatite-coated titanium implants. Biomaterials.

[49] Poth N., Seiffart V., Gross G., Menzel H., Dempwolf W. (2015). Biodegradable chitosan nanoparticle coatings on titanium for the delivery of BMP-2. Biomolecules.

[50] Kazek-Kęsik A., Nosol A., Płonka J., Śmiga-Matuszowicz M., Gołda-Cępa M., Krok-Borkowicz M. (2019). PLGA-amoxicillin-loaded layer formed on anodized Ti alloy as a hybrid material for dental implant applications. Mater. Sci. Eng.: C.

[51] Shi S., Cheng X., Wang J., Zhang W., Peng L., Zhang Y. (2009). RhBMP-2 microspheres-loaded chitosan/collagen scaffold enhanced osseointegration: an experiment in dog. J. Biomater. Appl..

[52] Van Der Stok J., Lozano D., Chai Y.C., Amin Yavari S., Bastidas Coral A.P., Verhaar J.A. (2015). Osteostatin-coated porous titanium can improve early bone regeneration of cortical bone defects in rats. Tissue Eng..

[53] Tejeda-Montes E., Smith K.H., Rebollo E., Gómez R., Alonso M., Rodriguez-Cabello J.C. (2014). Bioactive membranes for bone regeneration applications: effect of physical and biomolecular signals on mesenchymal stem cell behavior. Acta Biomater..

[54] Mathew A., Vaquette C., Hashimi S., Rathnayake I., Huygens F., Hutmacher D.W., Ivanovski S. (2017). Antimicrobial and immunomodulatory surface-functionalized electrospun membranes for bone regeneration. Adv. Healthc. Mater..

[55] Mirzaeei S., Mansurian M., Asare-Addo K., Nokhodchi A. (2021). Metronidazole-and amoxicillin-loaded PLGA and PCL nanofibers as potential drug delivery systems for the treatment of periodontitis: in vitro and in vivo evaluations. Biomedicines.

[56] Liu L.T., Chen M.J., Yang H.L., Huang Z.J., Tang Q., Chow C.F. (2020). An NIR-light-responsive surface molecularly imprinted polymer for photoregulated drug release in aqueous solution through porcine tissue. Mater. Sci. Eng.: C.

[57] Dhavale R.P., Dhavale R.P., Sahoo S.C., Kollu P., Jadhav S.U., Patil P.S. (2020). Chitosan coated magnetic nanoparticles as carriers of anticancer drug Telmisartan: pH-responsive controlled drug release and cytotoxicity studies. J. Phys. Chem. Solid..

[58] Yin X., Li Y., Yang C., Weng J., Wang J., Zhou J., Feng B. (2019). Alginate/chitosan multilayer films coated on IL-4-loaded TiO2 nanotubes for modulation of macrophage phenotype. Int. J. Biol. Macromol..

[59] Paris J.L., Román J., Manzano M., Cabañas M.V., Vallet-Regí M. (2015). Tuning dual-drug release from composite scaffolds for bone regeneration. Int. J. Pharmaceut..

[60] Kantarci A., Hasturk H., Van Dyke T.E. (2000). Animal models for periodontal regeneration and peri-implant responses. Periodontol.

[61] Kolenbrander P.E., Palmer R.J., Periasamy S., Jakubovics N.S. (2010). Oral multispecies biofilm development and the key role of cell–cell distance. Nat. Rev. Microbiol..

[62] Franca C.M., de Souza Balbinot G., Cunha D., Saboia V.D.P.A., Ferracane J., Bertassoni L.E. (2022). In-vitro models of biocompatibility testing for restorative dental materials: from 2D cultures to organs on-a-chip. Acta Biomater..

[63] Hadjichristou C., Papachristou E., Bonovolias I., Bakopoulou A. (2020). A. Three-dimensional tissue engineering-based Dentin/Pulp tissue analogue as advanced biocompatibility evaluation tool of dental restorative materials. Dent. Mater..

[64] Bhatia S.N., Ingber D.E. (2014). Microfluidic organs-on-chips. Nat. biotechnol..

[65] Ribitsch I., Baptista P.M., Lange-Consiglio A., Melotti L., Patruno M., Jenner F. (2020). Large animal models in regenerative medicine and tissue engineering: to do or not to do. Front. Bioeng. Biotechnol..

[66] Muschler G.F., Raut V.P., Patterson T.E., Wenke J.C., Hollinger J.O. (2010). The design and use of animal models for translational research in bone tissue engineering and regenerative medicine. Tissue Eng. B Rev..

[67] Schafrum Macedo A., Cezaretti Feitosa C., Yoiti Kitamura Kawamoto F., Vinicius Tertuliano Marinho P., dos Santos Dal-Bó I., Fiuza Monteiro B. (2019). Animal modeling in bone research—should we follow the White Rabbit?. Anim. Models Exp. Medic..

[68] Liu J., Xu H., Tang X., Xu J., Jin Z., Li H. (2017). Simple and tunable surface coatings via polydopamine for modulating pharmacokinetics, cell uptake and biodistribution of polymeric nanoparticles. RSC Adv..

[69] Tian A., Zhai J.J., Peng Y., Zhang L., Teng M.H., Liao J. (2014). Osteoblast response to titanium surfaces coated with strontium ranelate–loaded chitosan film. Int. J. Oral Maxillofac. Implants.

[70] Memarzadeh K., Sharili A.S., Huang J., Rawlinson S.C., Allaker R.P. (2015). Nanoparticulate zinc oxide as a coating material for orthopedic and dental implants. J. Biomed. Mater. Res., Part A.

[71] Rahmati M., Lyngstadaas S.P., Reseland J.E., Andersbakken I., Haugland H.S., López-Peña M. (2020). Coating doxycycline on titanium-based implants: two in vivo studies. Bioact. Mater..

[72] Valic M.S., Zheng G. (2019). Research tools for extrapolating the disposition and pharmacokinetics of nanomaterials from preclinical animals to humans. Theranostics.

[73] Abtahi J., Henefalk G., Aspenberg P. (2019). Impact of a zoledronate coating on early post-surgical implant stability and marginal bone resorption in the maxilla—a split-mouth randomized clinical trial. Clin. Oral Implants Res..

[74] Jensen O.T., Kuhlke K.L., Leopardi A., Adams M.W., Ringeman J.L. (2014). BMP-2/ACS/allograft for combined maxillary alveolar split/sinus floor grafting with and without simultaneous dental implant placement: report of 21 implants placed into 7 alveolar split sites followed for up to 3 years. Int. J. Oral Maxillofac. Implants.

[75] Alraei K., Shrqawi J., Alarusi K. (2021). Application of recombinant human BMP-2 with bone marrow aspirate concentrate and platelet-rich fibrin in titanium mesh for vertical maxillary defect reconstruction prior to implant placement. Case Rep. Dent..

[76] Lerouge S. (2019). Metals for Biomedical Devices.

[77] Baldin E.K.K., Malfatti C.D.F., Rodói V., Brandalise R.N. (2019). Effect of sterilization on the properties of a bioactive hybrid coating containing hydroxyapatite. Adv. Mater. Sci. Eng..

[78] Babaei S., Kasimov A., Girard-Lauriault P.L. (2018). The effect of sterilization procedures on the physiochemical properties and performance of plasma polymer films. Plasma Process. Polym..

[79] França A., Pelaz B., Moros M., Sánchez-Espinel C., Hernández A., Fernández-López C. (2010). Sterilization matters: consequences of different sterilization techniques on gold nanoparticles. Small.

[80] Guo T., Oztug N.A.K., Han P., Ivanovski S., Gulati K. (2021). Influence of sterilization on the performance of anodized nanoporous titanium implants. Mater. Sci. Eng.: C.

[81] Hasirci V., Hasirci N. (2018). Fundamentals of Biomaterials.

[82] Kunrath M.F., Vargas A.L., Sesterheim P., Teixeira E.R., Hubler R. (2020). Extension of hydrophilicity stability by reactive plasma treatment and wet storage on TiO2 nanotube surfaces for biomedical implant applications. J. R. Soc. Interface.

[83] Zinelis S., Silikas N., Thomas A., Syres K., Eliades G. (2012). Surface characterization of SLActive® dental implants. Eur. J. Esthetic Dent..

[84] Maulvi F.A., Choksi H.H., Desai A.R., Patel A.S., Ranch K.M., Vyas B.A., Shah D.O. (2017). pH triggered controlled drug delivery from contact lenses: addressing the challenges of drug leaching during sterilization and storage. Colloids Surf. B Biointerfaces.

[85] Eliaz N. (2019). Corrosion of metallic biomaterials: a review. Materials.

[86] Chadha R., Kashid N., Jain D.V.S. (2003). Kinetic studies of the degradation of an aminopenicillin antibiotic (amoxicillin trihydrate) in aqueous solution using heat conduction microcalorimetry. J. Pharm. Pharmacol..

[87] Vahdat L., Sunderland V.B. (2007). Kinetics of amoxicillin and clavulanate degradation alone and in combination in aqueous solution under frozen conditions. Int. J. Pharm. (Amst.).

[88] Esteban S.L., Manzo R.H., Alovero F.L. (2009). Azithromycin loaded on hydrogels of carbomer: chemical stability and delivery properties. Int. J. Pharm. (Amst.).

[89] Oliveira A.L., Pedro A.J., Arroyo C.S., Mano J.F., Rodriguez G., Roman J.S., Reis R.L. (2010). Biomimetic Ca-P coatings incorporating bisphosphonates produced on starch-based degradable biomaterials. J. Biomed. Mater. Res. Part B: Appl Biomater.

[90] Fazil M., Baboota S., Sahni J.K., Ameeduzzafar, Ali J. (2015). Bisphosphonates: therapeutics potential and recent advances in drug delivery. Drug Deliv..

[91] Guillot R., Gilde F., Becquart P., Sailhan F., Lapeyrere A., Logeart-Avramoglou D., Picart C. (2013). The stability of BMP loaded polyelectrolyte multilayer coatings on titanium. Biomaterials.

[92] Eawsakul K., Tancharoen S., Nasongkla N. (2021). Combination of dip coating of BMP-2 and spray coating of PLGA on dental implants for osseointegration. J. Drug Deliv. Sci. Technol..

[93] Zhou J., Guo X., Zheng Q., Wu Y., Cui F., Wu B. (2017). Improving osteogenesis of three-dimensional porous scaffold based on mineralized recombinant human-like collagen via mussel-inspired polydopamine and effective immobilization of BMP-2-derived peptide. Colloids Surf. B Biointerfaces.

[94] Safi S., Karimzadeh F., Labbaf S. (2018). Mesoporous and hollow hydroxyapatite nanostructured particles as a drug delivery vehicle for the local release of ibuprofen. Mater. Sci. Eng.: C.

[95] Li L., Xie C., Xiao X. (2020). Polydopamine modified TiO2 nanotube arrays as a local drug delivery system for ibuprofen. J. Drug Deliv. Sci. Technol..

[96] Rubasinghege G., Gurung R., Rijal H., Maldonado-Torres S., Chan A., Acharya S. (2018). Abiotic degradation and environmental toxicity of ibuprofen: roles of mineral particles and solar radiation. Water Res..

[97] Serri C., de Gennaro B., Quagliariello V., Iaffaioli R.V., De Rosa G., Catalanotti L. (2017). Surface modified zeolite-based granulates for the sustained release of diclofenac sodium. Eur. J. Pharmaceut. Sci..

[98] Toński M., Dołżonek J., Stepnowski P., Białk-Bielińska A. (2019). Hydrolytic stability of selected pharmaceuticals and their transformation products. Chemosphere.

[99] Duddeck D.U., Albrektsson T., Wennerberg A., Larsson C., Beuer F. (2019). On the cleanliness of different oral implant systems: a pilot study. J. Clin. Med..

[100] Penha N., Groisman S., Ng J., Gonçalves O.D., Kunrath M.F. (2018). Physical-chemical analyses of contaminations and internal holes in dental implants of pure commercial titanium. J. Osseointegr..

[101] Li T., Gulati K., Wang N., Zhang Z., Ivanovski S. (2018). Understanding and augmenting the stability of therapeutic nanotubes on anodized titanium implants. Mater. Sci. Eng. C.

[102] Verissimo T.V., Santos N.T., Silva J.R., Azevedo R.B., Gomes A.J., Lunardi C.N. (2016). In vitro cytotoxicity and phototoxicity of surface-modified gold nanoparticles associated with neutral red as a potential drug delivery system in phototherapy. Mater. Sci. Eng.: C.

[103] Panzarini E., Mariano S., Carata E., Mura F., Rossi M., Dini L. (2018). Intracellular transport of silver and gold nanoparticles and biological responses: an update. Int. J. Mol. Sci..

[104] Asa’ad F., Thomsen P., Kunrath M.F. (2022). The role of titanium particles and ions in the pathogenesis of peri-implantitis. J. Bone Metabol..

[105] Kunrath M.F., Muradás T.C., Penha N., Campos M.M. (2021). Innovative surfaces and alloys for dental implants: what about biointerface-safety concerns?. Dent. Mater..

[106] Bush K., Courvalin P., Dantas G., Davies J., Eisenstein B., Huovinen P. (2011). Tackling antibiotic resistance. Nat. Rev. Microbiol..

[107] Truebel H., Thurston T. (2020).

[108] Chen Y.C., Gad S.F., Chobisa D., Li Y., Yeo Y. (2021). Local drug delivery systems for inflammatory diseases: status quo, challenges, and opportunities. J. Contr. Release.

[109] Wei Y., Deng Y., Ma S., Ran M., Jia Y., Meng J. (2021).

[110] Klinge B., Lundström M., Rosén M., Bertl K., Klinge A., Stavropoulos A. (2018). Dental implant quality Register—a possible tool to further improve implant treatment and outcome. Clin. Oral Implants Res..

[111] Moraschini V., Poubel L.D.C., Ferreira V.F., dos Sp Barboza E. (2015). Evaluation of survival and success rates of dental implants reported in longitudinal studies with a follow-up period of at least 10 years: a systematic review. Int. J. Oral Maxillof. Surg..

[112] Lim G., Lin G.H., Monje A., Chan H.L., Wang H.L. (2018). Wound healing complications following guided bone regeneration for ridge augmentation: a systematic review and meta-analysis. Int. J. Oral Maxillofac. Implants.

[113] Pjetursson B.E., Thoma D., Jung R., Zwahlen M., Zembic A. (2012). A systematic review of the survival and complication rates of implant-supported fixed dental prostheses (FDPs) after a mean observation period of at least 5 years. Clin. Oral Implants Res..

[114] Research Report Dental Implants Market (2020). https://www.fortunebusinessinsights.com/industry1448%20reports/dental-implants-market-100443.

[115] Hasan J., Bright R., Hayles A., Palms D., Zilm P., Barker D., Vasilev K. (2022). Preventing peri-implantitis: the quest for a next generation of titanium dental implants. ACS Biomater. Sci. Eng..

[116] (2022). Novel Drug Delivery Systems in Cancer Therapy Market Report.

[117] Stearns L.J., Narang S., Albright R.E., Hammond K., Xia Y., Richter H.B. (2019). Assessment of health care utilization and cost of targeted drug delivery and conventional medical management vs conventional medical management alone for patients with cancer-related pain. JAMA Netw. Open.

[118] Geethakumari P.R., Ramasamy D.P., Dholaria B., Berdeja J., Kansagra A. (2021). Balancing quality, cost, and access during delivery of newer cellular and immunotherapy treatments. Curr. Hematol. Malign. Rep..

[119] Dawidczyk C.M., Kim C., Park J.H., Russell L.M., Lee K.H., Pomper M.G., Searson P.C. (2014). State-of-the-art in design rules for drug delivery platforms: lessons learned from FDA-approved nanomedicines. J. Contr. Release.

[120] Zolnik B.S., Sadrieh N. (2009). Regulatory perspective on the importance of ADME assessment of nanoscale material containing drugs. Adv. Drug Deliv. Rev..

[121] Ragelle H., Danhier F., Préat V., Langer R., Anderson D.G. (2017). Nanoparticle-based drug delivery systems: a commercial and regulatory outlook as the field matures. Expet Opin. Drug Deliv..

[122] Hassan S., Prakash G., Ozturk A.B., Saghazadeh S., Sohail M.F., Seo J. (2017). Evolution and clinical translation of drug delivery nanomaterials. Nano Today.

[123] Ezzat S.M., El Bishbishy M.H., Walag A.M.P., Mtewa A.G. (2020). Phytochemicals as Lead Compounds for New Drug Discovery.

[124] Pokrowiecki R., Szałaj U., Fudala D., Zaręba T., Wojnarowicz J., Łojkowski W. (2022). Dental implant healing screws as temporary oral drug delivery systems for decrease of infections in the area of the head and neck. Int. J. Nanomed..

[125] Kwon D.H., Lee S.J., Wikesjö U.M., Johansson P.H., Johansson C.B., Sul Y.T. (2017). Bone tissue response following local drug delivery of bisphosphonate through titanium oxide nanotube implants in a rabbit model. J. Clin. Periodontol..

[126] Caplin J.D., García A.J. (2019). Implantable antimicrobial biomaterials for local drug delivery in bone infection models. Acta Biomater..

[127] Uskokovic V. (2015). Nanostructured platforms for the sustained and local delivery of antibiotics in the treatment of osteomyelitis. Crit. Rev. Ther. Drug Carrier Syst..

[128] Fabio Oliveira de Sousa F., Ferraz C., de Santiago Nojosa J., Yamauti M. (2014). Nanotechnology in dentistry: drug delivery systems for the control of biofilm-dependent oral diseases. Curr. Drug Deliv..

[129] Nastri L., De Rosa A., De Gregorio V., Grassia V., Donnarumma G. (2019). A new controlled-release material containing metronidazole and doxycycline for the treatment of periodontal and peri-implant diseases: formulation and in vitro testing. Int. J. Dent..

[130] Talebi Bezmin Abadi A., Rizvanov A.A., Haertlé T., Blatt N.L. (2019). World Health Organization report: current crisis of antibiotic resistance. BioNanoScience.

[131] Costa J.V., Portugal J., Neves C.B., Bettencourt A.F. (2021). Should local drug delivery systems be used in dentistry?. Drug Deliv. Translat. Res..

[132] Lal A., Alam M.K., Ahmed N., Maqsood A., Al-Qaisi R.K., Shrivastava D. (2021). Nano drug delivery platforms for dental application: infection control and TMJ management—a review. Polymers.

[133] Kunrath M.F. (2020). Customized dental implants: manufacturing processes, topography, osseointegration and future perspectives of 3D fabricated implants. Bioprinting.

[134] Pommer B., Zechner W., Watzak G., Ulm C., Watzek G., Tepper G. (2011). Progress and trends in patients' mindset on dental implants. II: implant acceptance, patient-perceived costs and patient satisfaction. Clin. Oral Implants Res..

[135] Esposito M., Grusovin M.G., Felice P., Karatzopoulos G., Worthington H.V., Coulthard P. (2010). The efficacy of horizontal and vertical bone augmentation procedures for dental implants: a Cochrane systematic review. Evid.-based Practice: Toward Optimiz. Clin. Outcomes.

[136] Campoccia D., Montanaro L., Arciola C.R., C. (2013). A review of the clinical implications of anti-infective biomaterials and infection-resistant surfaces. Biomaterials.

[137] Eloy R. (2012). Biocompatibility and Performance of Medical Devices.

